# Clinical performance of long axial field of view PET/CT: a head-to-head intra-individual comparison of the Biograph Vision Quadra with the Biograph Vision PET/CT

**DOI:** 10.1007/s00259-021-05282-7

**Published:** 2021-04-02

**Authors:** Ian Alberts, Jan-Niklas Hünermund, George Prenosil, Clemens Mingels, Karl Peter Bohn, Marco Viscione, Hasan Sari, Bernd Vollnberg, Kuangyu Shi, Ali Afshar-Oromieh, Axel Rominger

**Affiliations:** 1 Department of Nuclear Medicine, Inselspital, Bern University Hospital, University of Bern, Freiburgstr. 18, 3010 Bern, Switzerland; 2 Advanced Clinical Imaging Technology, Siemens Healthcare AG, Lausanne, Switzerland

**Keywords:** Total-body, Ultra-long FOV PET, Whole-body, PET/CT, Positron-emission-tomography, Digital PET

## Abstract

**Purpose:**

To investigate the performance of the new long axial field-of-view (LAFOV) Biograph Vision Quadra PET/CT and a standard axial field-of-view (SAFOV) Biograph Vision 600 PET/CT (both: Siemens Healthineers) system using an intra-patient comparison.

**Methods:**

Forty-four patients undergoing routine oncological PET/CT were prospectively included and underwent a same-day dual-scanning protocol following a single administration of either ^18^F-FDG (*n* = 20), ^18^F-PSMA-1007 (*n* = 16) or ^68^Ga-DOTA-TOC (*n* = 8). Half the patients first received a clinically routine examination on the SAFOV (FOV_axial_ 26.3 cm) in continuous bed motion and then immediately afterwards on the LAFOV system (10-min acquisition in list mode, FOV_axial_ 106 cm); the second half underwent scanning in the reverse order. Comparisons between the LAFOV at different emulated scan times (by rebinning list mode data) and the SAFOV were made for target lesion integral activity, signal to noise (SNR), target lesion to background ratio (TBR) and visual image quality.

**Results:**

Equivalent target lesion integral activity to the SAFOV acquisitions (16-min duration for a 106 cm FOV) were obtained on the LAFOV in 1.63 ± 0.19 min (mean ± standard error). Equivalent SNR was obtained by 1.82 ± 1.00 min LAFOV acquisitions. No statistically significant differences (*p* > 0.05) in TBR were observed even for 0.5 min LAFOV examinations. Subjective image quality rated by two physicians confirmed the 10 min LAFOV to be of the highest quality, with equivalence between the LAFOV and the SAFOV at 1.8 ± 0.85 min. By analogy, if the LAFOV scans were maintained at 10 min, proportional reductions in applied radiopharmaceutical could obtain equivalent lesion integral activity for activities under 40 MBq and equivalent doses for the PET component of <1 mSv.

**Conclusion:**

Improved image quality, lesion quantification and SNR resulting from higher sensitivity were demonstrated for an LAFOV system in a head-to-head comparison under clinical conditions. The LAFOV system could deliver images of comparable quality and lesion quantification in under 2 min, compared to routine SAFOV acquisition (16 min for equivalent FOV coverage). Alternatively, the LAFOV system could allow for low-dose examination protocols. Shorter LAFOV acquisitions (0.5 min), while of lower visual quality and SNR, were of adequate quality with respect to target lesion identification, suggesting that ultra-fast or low-dose acquisitions can be acceptable in selected settings.

## Introduction

Hybrid nuclear medicine and molecular imaging has undergone much development since the first clinical introduction of positron emission tomography/computed tomography (PET/CT) at the turn of the twenty-first century [1]. For example, recently introduced PET systems with silicon photomultipliers (SiPM) surmount a number of limitations encountered with previous generation scanners based on photomultiplier tubes. Such fully digital PET/CT systems offer a number of technical and clinical advantages [2, 3], with corresponding improvements in image quality and lesion detection [3–9]. Although such systems have included longer axial coverage compared to previous generation systems, typically less than an eighth of the body can be examined in the field of view (FOV) at a given time. As a result, less than 1% of all emitted coincidence photons can be detected, placing inherent physical limits on the detection efficiency and sensitivity of such systems [10].

Most recently, long axial field-of-view (LAFOV) scanners with SiPM detection systems have been introduced, with Badawi et al. reporting the first clinical experiences with a 194-cm FOV scanner (uExplorer, United Imaging Healthcare Co, Shanghai, China) [11], which provided substantially improved count density when compared with previous-generation standard axial field-of-view (SAFOV) systems. The signal-to-noise ratio (SNR) of a PET system is given by the well-known relationship, where *k* is a constant (which also include a gain factor due to the use of time-of-flight), *S* is the sensitivity of the scanner, *A*_R_ is the applied radiopharmaceutical activity and *T*_a_ the total acquisition time:

$$ \mathrm{SNR}\approx k\sqrt{S\times {A}_{\mathrm{R}}\times {T}_{\mathrm{a}}} $$

As such, the improved sensitivity can improve SNR, or allow for reductions in applied radiopharmaceutical activities or shorter duration acquisitions while providing equivalent image quality. Although the performance of the pioneering uEXPLORER LAFOV system has been extensively characterised by phantom measurements [12], the performance and utility of such LAFOV systems have been less well evaluated in clinical settings so far. The first Biograph Vision Quadra PET/CT LAFOV system worldwide (“Quadra”, Siemens Healthineers, Knoxville, TN, USA) with a FOV of 106 cm was installed in October 2020 at the Department for Nuclear Medicine, Inselspital, University Hospital Bern, in Switzerland. Preliminary assessments of this scanner’s characteristics reveal a sensitivity of 174 cps/kBq and a time of flight (TOF) resolution of 219 ps in ultra-high sensitivity mode [13]. The aim of this study is to provide an intra-individual comparison of this novel LAFOV system with the clinically well-established Biograph Vision 600 (Siemens Healthineers, Knoxville, TN, USA) SAFOV system, with a standard axial FOV of 26.3 cm. To the best of our knowledge, this study represents the first intra-individual head-to-head comparison of an LAFOV with a SAFOV in a clinical setting as well as the first clinical experiences with this new LAFOV system.

## Materials and methods

### Study design

This prospective, non-randomised, dual-arm crossover, comparative imaging study aims to compare the subjective imaging quality by an intra-individual comparison of scans obtained on a SAFOV and a LAFOV system. The primary objective is to estimate the scan time on the LAFOV system (0.5 to 10 min) giving equivalent count statistics to routine clinical examinations on the SAFOV system, and thereby quantifying the benefit of LAFOV systems in terms of lesion quantification in a clinical setting. The secondary objectives are to compare image quality in terms of image signal-to-noise ratio (SNR), subjective image quality as rated by clinicians and target lesion signal to background noise ratios (TBR). The study hypothesis was that, with a LAFOV system, one can achieve superior lesion quantification in terms of integral measured activity compared to a standard-of-care acquisition on a SAFOV. The endpoint of the study was the exposure time on the LAFOV yielding target lesion integral measured activity equivalent to the SAFOV.

**Fig. 1:**
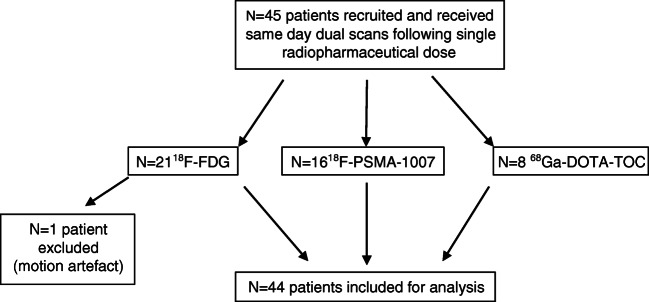
Study flowchart showing patient recruitment, total patients included and excluded

We assume a minimum effect size of ±1 min equivalent scan time and a pragmatic estimate for the standard deviation of ±1 min. For a two tailed *α* = 0.05 and a study power of 85%, a target sample size of *n* > 40 individuals based on the t-statistic and non-centrality parameter was calculated. Inclusion criteria were individuals over the age of 18 willing to undergo a second examination and with at least one positive target lesion in the first examination. Exclusion criteria were lack of target lesions in the first PET/CT, inability to provide informed consent, claustrophobia or inability to undergo a second examination. One patient undergoing ^18^F-FDG-PET/CT was excluded from the study owing to significant motion artefact during the second examination; all other patients were included in the analysis. The study flowchart is in Fig. 1. This prospective study was approved by the regional ethics committee (KEK 2020/01413) and performed in accordance with the declaration of Helsinki and all relevant national legislation.

### Patient population

Target recruitment was met with recruitment of 44 individuals undergoing clinically routine oncological PET/CT at the University Hospital Bern (*n* = 20 with ^18^F-FDG, *n* = 16 ^18^F-PSMA-1007, *n* = 8 ^68^Ga-DOTA-TOC) between 10 and 12/2020. Patients undergoing ^18^F-FDG studies were selected to provide a broad and balanced selection of tumour entities, and patients undergoing ^18^F-PSMA-1007 and ^68^Ga-DOTA-TOC were referred for examination of known prostate cancer (*n* = 13 biochemical recurrence and *n* = 1 histologically confirmed primary prostate cancer) or histologically confirmed SSTR-2 positive neuroendocrine tumours respectively. All patients provide written informed consent for inclusion in this study, which was approved by the regional ethics committee.

### Imaging routines

As per clinical routine, we required patients referred for ^18^F-FDG studies to have fasted for >6 h prior to scanning and a venous blood glucose of <120 mg/dl was confirmed by finger-prick sampling. Patients undergoing ^18^F-PSMA-1007 and ^68^Ga-DOTA-TOC studies did not require specific preparation. Standardised doses of 3.5 MBq/kg of [^18^F]-FDG, 250 MBq ^18^F-PSMA-1007 and 150 MBq ^68^Ga-DOTA-TOC were administered intravenously. Scans were acquired at 60 min post-injection of radiotracer (p.i.) for ^18^F-FDG and 120 min for ^68^Ga-DOTA-TOC and ^18^F-PSMA-1007. All patients received regular whole-body PET scans (from skull-base to the thighs) on either the SAFOV scanner (Biograph Vision) or vertex to thighs on the LAFOV scanner (Biograph Vision Quadra).

Patients were divided into equal groups receiving successive, same-day examination with no further radiopharmaceutical applied. The first group underwent initial examination on the Biograph Vision and then an additional examination on the Biograph Vision Quadra; the second group underwent these examinations in the reverse order.

### Imaging protocol

Whole body PET images were reconstructed with the same reconstruction parameters for both systems in 3D with a zoom factor of 1.0. Emission data were corrected for randoms, scatter and decay, and reconstruction was with the vendor’s time of flight (TOF) point-spread-function (PSF) algorithm with 4 iterations and 5 subsets. A Gauss filter was applied (2 mm FWHM). Images were reconstructed to 440 × 440 × 644 image matrix with a voxel size of 1.65 × 1.65 × 1.65 mm^3^. Attenuation correction was performed using the low-dose non-enhanced computed tomography data. Images were acquired on the SAFOV in continuous bed motion (CBM) with a table velocity of 1.1 mm/s, equivalent to 2 min/bed position (bp) (https://www.siemens-healthineers.com/molecular-imaging/options-and-upgrades/software-applications/flowmotion-technology). Consequently, an effective examination time of 16.06 min would be required to capture an equivalent 106 cm FOV in CBM at a velocity of 1.1 mm/s, where the 2 min/bp includes table overlap. Images were acquired on the LAFOV in one bed position for a total acquisition of 10 min. PET-data for the LAFOV were sampled to produce sinograms corresponding to emulate 10, 6, 4, 2, 1 and 0.5 min acquisitions. The LAFOV system was used with a maximum ring difference (MRD) of 85; the ultra-high sensitivity mode with a MRD of 322 was not available at time of this study.

CT scans were performed with equivalent parameters for both scanners with slice thickness of 1.0 mm, pitch factor 1, bone and soft tissue reconstruction kernels and maximum of 120 kV and 90 mAs by applying CARE kV and CARE Dose. Contrast-enhanced scans were acquired during the first clinically indicated and routine examination whenever clinically required.

### Image evaluation

Two experienced nuclear medicine physicians, who read all images in consensus, performed quantitative image evaluation and analysis. Both physicians using clinically established reading applications (*syngo*.via MMOncology; Siemens Healthineers, Erlangen, Germany) identified target lesions. Lesion uptake and metabolic tumour volumes were calculated by placing a volume-of-interest (VOI) around the lesion with 40% iso-contour approach as previously described [14]. Peak lesion activity and SUV_peak_ was used to evaluate target lesions, and this parameter has been shown to be less sensitive to acquisition time than SUV_max_ [15]. Lesions evaluated at different emulated scan times (obtained by binning the list mode data with different frame durations) for the LAFOV, and for the routine SAFOV acquisitions, were evaluated by comparison of lesion signal or integral measured activity: $A={\int}_0^t{A}_{\mathrm{l}}{T}_{LM}\  dt$ where *A*_l_ is lesion activity concentration in Bq/ml (where activities were decay corrected to time of injection) and *T*_*LM*_ is the acquisition duration in seconds (s), where scan acquisition duration is given by the time/bp). This lesion integral activity can be considered a measure of the count statistic or count density (counts/ml) [16]. A linear regression model was used for the LAFOV integral activity as a function of total acquisition duration, to calculate an equivalent scan time to yield equal integral lesion activity on the SAFOV, i.e. the time on the LAFOV giving equal count statistics to the SAFOV.

By maintaining the LAFOV examination at 10 min, instead of reduction in acquisition time, emulated reduction in radiopharmaceutical activities could be calculated for images of equivalent quality/count statistic to the SAFOV. The corresponding radiopharmaceutical dose equivalents were as follows: 0.019 mSv/MBq for ^18^F-FDG [17], 0.022 mSv/MBq for ^18^F-PSMA-1007 [18] and 0.023 mSv/MBq for ^68^Ga-DOTA-TOC [19].

The background was measured by the placement of a 14 cm^3^ volume-of-interest (VOI) in healthy liver tissue in the right liver lobe as previously described [17]. VOIs were copied and pasted between different images obtained from different (list mode) frame durations, ensuring that the same VOI was analysed for each acquisition which was confirmed by comparison of the metabolic tumour volume (MTV). A SNR was defined as the reciprocal coefficient of variation (COV) for the liver background (μ/σ), where *σ* = standard deviation of the background VOI and *μ* = background SUV_mean_ [20]. Lesion quantification was determined by calculating SUV_peak_, and tumour-to-background ratios (TBRs) were defined as SUV_peak_ of the target lesion divided by SUV_mean_ for the liver background. Finally, covariate analysis was performed for differences in uptake time (time window between first and second scan) and measured differences in measured integral activity.

### Image quality

An experienced nuclear medicine physician obtained maximum intensity projections (MIP) and example axial slices at the level of the bifurcation of the hepatic portal vein for each patient from both scanners (10, 4, 2, 1 and 0.5 min reconstructions for the LAFOV and the full-length acquisition for the SAFOV system). Images captured for each patient from both scanners were presented in randomised order and anonymised with respect to patient demographics, time of acquisition and scanner type. Images were cropped to include only “eyes-to-thighs” to minimise differences between captured FOV in both systems.

Two different nuclear medicine physicians who had no prior familiarity with the cases or analysis reviewed all images in consensus. Readers were blinded to scanner type and order of acquisition and DICOM data were not available to the readers studying these secondary captures. Using a Likert-scale, MIP images and axial slices for LAFOV and SAFOV images were ranked in order of quality (from highest to lowest). Where images were of equal quality, this was noted, and where the SAFOV scanner was ranked as intermediate between two scan times (e.g. as between a 1 and 2 min scan time) then the intermediate value was interpolated. In this way, the emulated scan time for the LAFOV giving equivalency in terms of subjective image quality to the SAFOV could be obtained. By analogy, images equivalent to the LAFOV scan could be obtained by maintaining the same LAFOV examination time (10 min) and proportional reduction in applied radiopharmaceutical activities (MBq), and the estimated effective radiation dose associated with this analogous scan (mSv).

### Statistical analysis

Statistical analyses and production of graphs were performed using Excel (Microsoft, Redmond, Washington) and R (version 4.0.3). Data are presented as mean ± standard error unless otherwise stated, with differences assessed by the paired Student’s *t* test. Linear regression analysis was used to obtain an equivalent scan time for the LAFOV giving equivalent integral activity or SNR to the SAFOV. Correlation between scan time and subjective image quality ranking for the LAFOV were compared by Spearman’s rank correlation coefficient. *p* values <0.05 were considered statistically significant and for multiple comparisons, the familywise error-rate was corrected for by using the Bonferroni method. Correlations between the time window between scans affecting lesion quantification co-variate analysis was performed using Pearson’s correlation coefficient *r*.

## Results

### Patient examination and target lesion detection

In total, 153 target lesions were identified in 44 patients. All target lesions were identified in both scanners. The mean radiopharmaceutical activity applied ± standard deviation, patient age, the effective radiation dose in mSv and the mean time window between the start of the first and the second scan are reported in Table 1. No statistically significant differences were observed for any of these parameters between the patients undergoing SAFOV as the first or second examination. The indications for the oncological PET/CT for the patients undergoing scanning with ^18^F-FDG are outlined in Table 1.

**Table 1:** Patient characteristics; *N* number of patients and by tumour type (*HCC* hepatocellular cancers, *ORL* head and neck cancers, *Colon ca* colorectal cancers); mSv equivalent dose PET component; mean radiopharmaceutical activity applied (MBq); ±SD standard deviation; age (years); mean delay (time window between start of the first examination and the start of the second examination)

Radiotracer	*N*, tumour type	mSV	Mean activity (MBq)	Mean activity SD	Age (a)	Mean Delay (hh:mm:ss)
^18^F-FDG	*N* = 20 (lung = 6, lymphoma 3, ORL 3, breast 2, thyroid 2, melanoma 2, HCC 1, Colon Ca 1	5.0	265.6	65.8	67.9	00:47:08
^18^F-PSMA-1007	*N* = 16 (14 biochemical recurrence, 2 primary)	5.4	243.9	14.0	75.5	00:57:21
^68^Ga-DOTA-TOC	*N* = 8 (SSTR expressing neuroendcrine tumours)	3.5	154.1	12.0	65.3	01:15:21

### Lesion integral activity

**Fig. 2:**
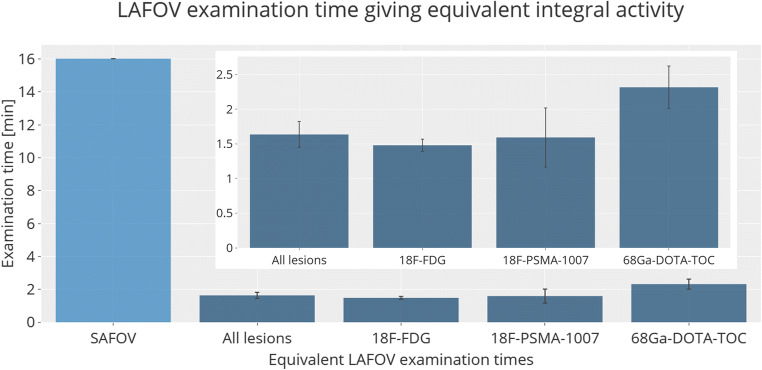
Scan times (min) for the LAFOV delivering equivalent lesion integral activity to the SAFOV standard examination (16.06 min). In the inset tile, a zoomed graph showing only LAFOV data is available to aid comparison. Error bars show the standard error

**Fig. 3:**
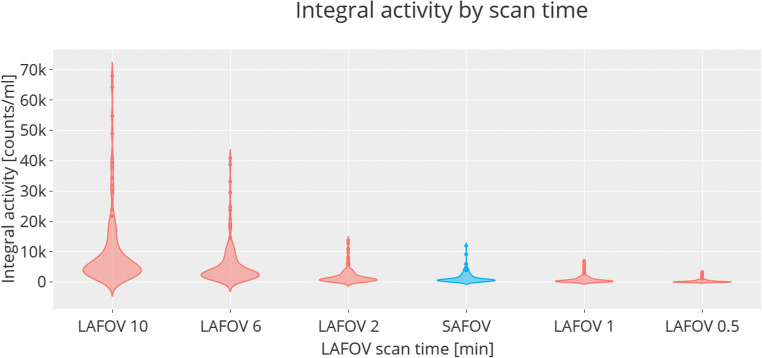
Violin plots showing lesion integral activity (all radiotracers) for the SAFOV (blue) and for various scan times (0.5 to 10 min) on the LAFOV (red). The measured integral activity on the SAFOV scanner was equivalent to between 1- and 2-min scans obtained on the LAFOV. The violin plots represent data density and distribution

The LAFOV scan that yielded equivalent target lesion integral activity (count statistic) to the SAFOV was 1.63 ± 0.19 min. Sub-group analysis by radiotracer is as follows: ^18^F-FDG 1.41 ± 1.01 min; ^18^F-PSMA-1007 1.59 ± 0.42 min; ^68^Ga-DOTA-TOC 2.32 ± 0.30 min (Fig. 2). The highest integral activities were obtained by the 10 min LAFOV scans, with data for all radiotracers at different emulated exposure times for the LAFOV shown in Fig. 3, visual inspection of which confirms the integral lesion activity on the SAFOV as being intermediate between the LAFOV 1 and 2 min acquisitions.

### Signal-to-noise ratio

**Fig. 4:**
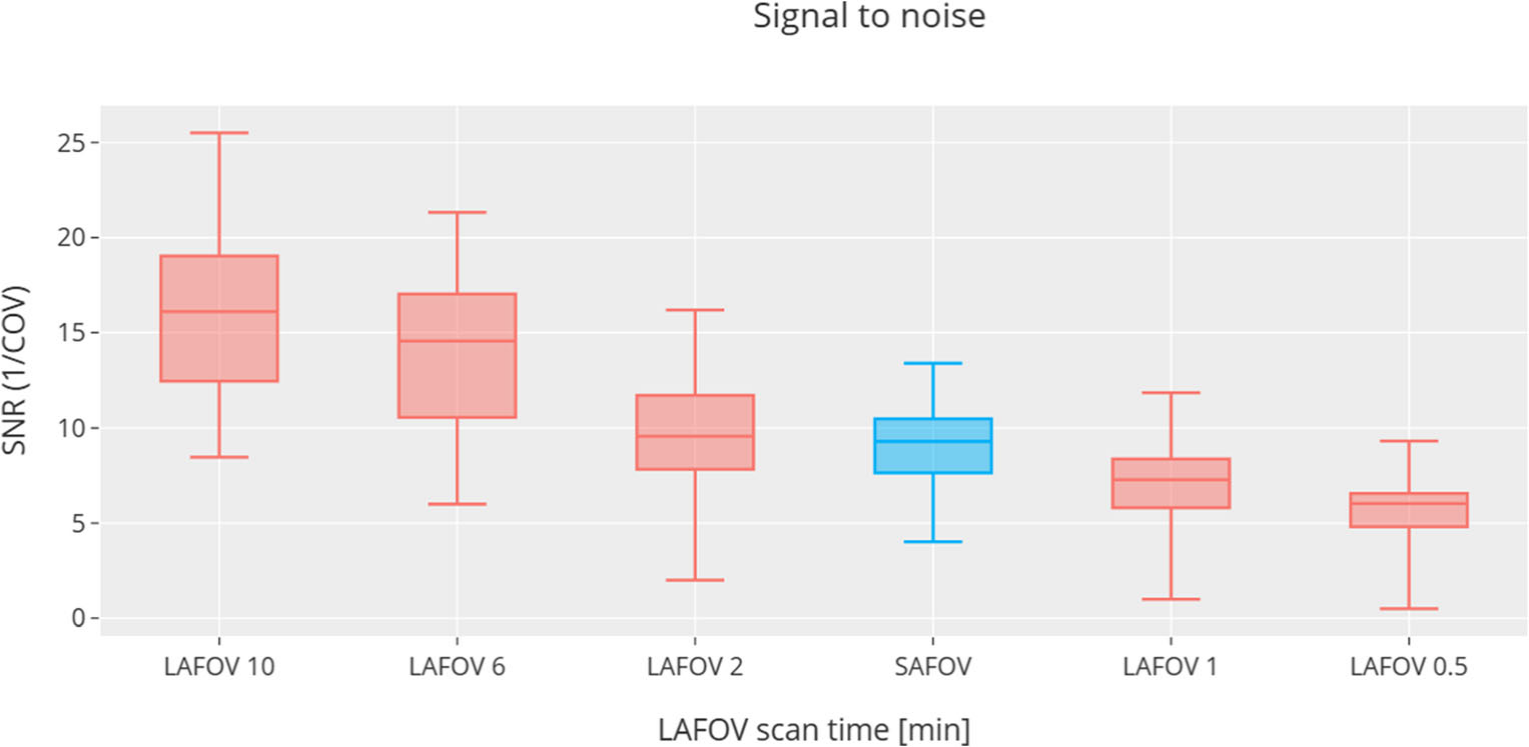
Boxplots showing signal-to-noise ratio (SNR) for the liver background, which is the reciprocal of the coefficient of variation. The measured SNR for SAFOV scanner (blue) was equivalent to between 1- and 2-min scans obtained on the LAFOV (red)

The SNR (inverse coefficient of variation) was highest for long-duration LAFOV images (10 min). Equivalency between the LAFOV and SAFOV was seen at 1.83 ± 1.00 min. The results are shown in Fig. 4.

### Target lesion-to-background ratio

**Fig. 5:**
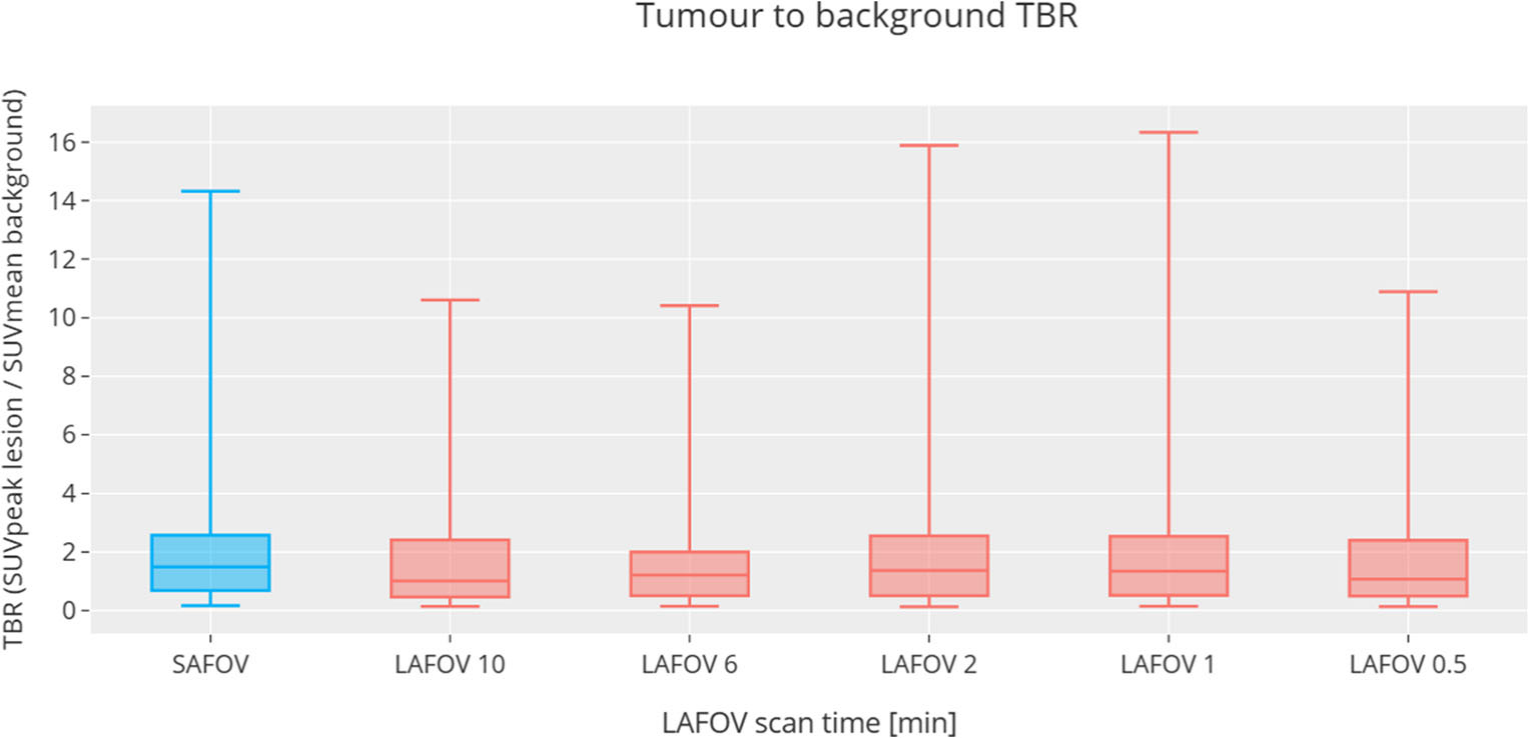
Tumour-to-background ratios (TBR) for the SAFOV (left, blue) and LAFOV (right, red). No statistically significant differences were observed between the SAFOV and LAFOV acquisitions, even at short (0.5 min) scan times

Improved TBR was seen on the LAFOV at 10 min (mean 2.27 ± 0.02) compared to the SAFOV (mean 2.06 ± 0.02). However, no statistically significant differences were observed for any LAFOV acquisition, even at 0.5 min, *p* = 0.47), suggesting adequate lesion-to-background contrast even for short acquisitions. The results are shown in Fig. 5.

### Image quality

**Fig. 6:**
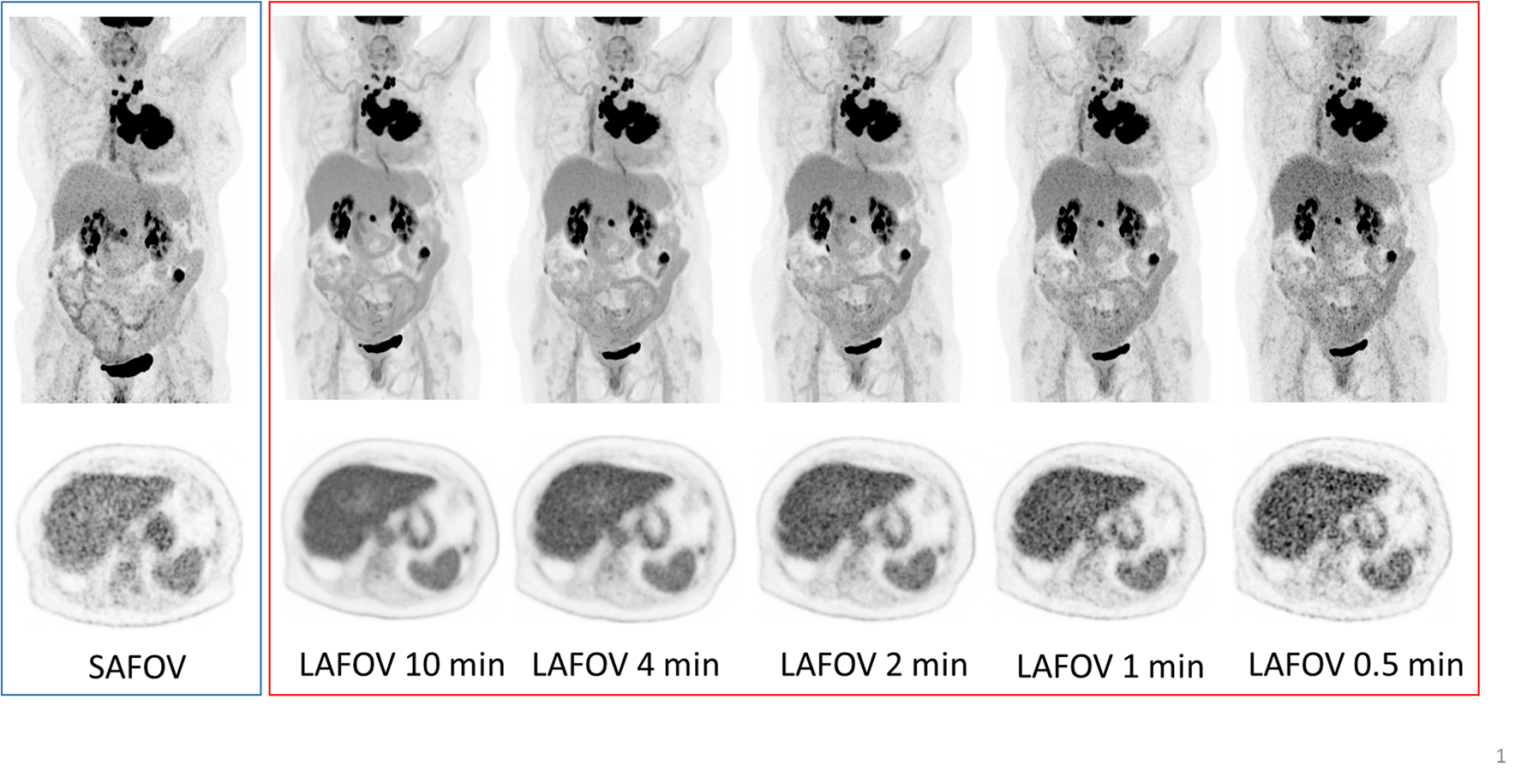
Example maximum intensity projection (MIP, top row) and axial PET images (bottom row) images for a 57-year-old female with non-small cell lung cancer, presented are images for the regular SAFOV acquisitions on the left (blue margin) and the LAFOV for 10-, 4-, 2-, 1- and 0.5-min acquisitions (right, red margin). For reference, the PET window is set to 0 to 8.5 SUV

All MIP and axial images (10, 4, 2, 1, 0.5 min LAFOV and full acquisition SAFOV) were ranked at blinded assessment in order of quality. The readers were unaware of the scanner type, scan order or clinical details and had not previously seen these cases. The reference SAFOV images were consistently ranked as of inferior quality, with a median ranking of 4th worst (range 3–5), and were largely evaluated as intermediate between the 2  and 0.5 min LAFOV images. Overall image quality on the LAFOV correlated with length of acquisition, with the 10 min being ranked as highest quality in 100% of the cases. The average scan times for the LAFOV ranked as equivalent to the SAFOV reference acquisitions were as follows: ^18^F-FDG 1.95 ± 0.86 min, ^18^F-PSMA-1007 1.95 ± 0.86 min, ^68^Ga-DOTA-TOC 1.50 ± 0.48 min. Image quality for the LAFOV correlated perfectly with length of acquisition time for all radiotracers (Pearson’s rank coefficient 0.997 for ^18^F-FDG, 1 for ^18^F-PSMA-1007 and ^68^Ga-DOTA-TOC). Example images are presented in Fig. 6.

Finally, while of consistently lower quality compared to the SAFOV reference images, the 0.5 min acquisitions on the LAFOV system provided visualisation of all target lesions and were of acceptable quality.

### Lesion quantification and time window between scans

The potential for the time between scans/imaging sequence to affect lesion quantification was considered. No statistically significant correlation was observed between difference in scan starting time and absolute difference in lesion peak integral activity between scan one and scan two (*r* = 0.17, *p* = 0.15) or for SNR (*r* = 0.39, *p* = 0.08).

### Equivalent low activity scan and equivalent radiation dose

Equivalent lesion measured integral activity compared to the SAFOV reference (15 min total scanning time in CBM) was achieved on the LAFOV with an average 1.63 min scan time with the whole FOV captured in one bed position (e.g. 16.06÷1.63 = 9.9x reduction in effective examination time). Alternatively, LAFOV examinations could be maintained at 10 min with proportional (e.g. 10÷1.63 = 6.1x) reduction in the injected radiopharmaceutical activity (where the product of applied radiopharmaceutical activity × acquisition time/bp is equal). The calculated equivalent activities and resultant radiation doses for each radiotracer are shown in Table 2.

**Table 2:** Equivalent acquisition times for equivalent target lesion integral activities obtained for the SAFOV (Vision) and LAFOV (Quadra) systems. Activities (MBq) and corresponding equivalent radiation dose (mSv) giving equivalent target lesion integral activity for examination times on the LAFOV equalling the SAFOV are given

	Examination time (min)		Equivalent activity (MBq)		Equivalent dose (mSv)	
Radiotracer	SAFOV	LAVFOV	SAFOV	LAFOV	SAFOV	LAFOV
^18^F-FDG	16.06	1.48	265.6	39.3	5.04	0.75
^18^F-PSMA-1007	16.06	1.59	243.9	38.8	5.37	0.85
^68^Ga-DOTA-TOC	16.06	2.32	154.1	35.7	3.54	0.82

## Discussion

This present study represents the first published clinical experiences with the Siemens Biograph Vision Quadra PET/CT system, and the first intra-individual comparative imaging study comparing a long axial FOV PET/CT scanner and a standard FOV, clinically established PET/CT system.

Previously published pioneering studies using LAFOV systems report increased detection efficiency, the potential for ultra-short or low-dose examination protocols and increased dynamic range of the scanners affording later image acquisitions [10, 11]. However, much of the hitherto published literature on LAFOV is limited to case studies as initial clinical experiences [11] or phantom studies [12]. Most recently, a dual-armed study with two cohorts randomised to either full (SAFOV) or half-dose scanning using a LAFOV system was published in a cohort of patients with lung cancer [21], and low-dose protocols have been examined in healthy volunteers [22]. However, the full potential of LAFOV systems is yet to be characterised in a clinical setting.

In this present study, we compared LAFOV images to a standard-of-care acquisition on a SAFOV scanner. The SAFOV acquisitions were obtained in CBM at a table velocity of 1.1 mm/s. For a SAFOV including overlap in bed positions to account for the loss of sensitivity at the extremes of the FOV [23], this table velocity is equivalent to a 2 min/bp exposure (https://www.siemens-healthineers.com/molecular-imaging/options-and-upgrades/software-applications/flowmotion-technology). An effective examination time of 16.06 min can be computed to obtain a FOV of 106 cm (“eyes to thighs”), which can be achieved in one bed position on the LAFOV. In this study, we demonstrate that equivalent lesion activity to the standard-of-care SAFOV acquisition is obtained by the LAFOV in 1.63 ± 0.19 min, i.e. a factor of 9.9x faster for equivalent count statistics. Such images were not at the detriment of image quality, with equivalence in image SNR being obtained at 1.82 ± 1.00 min. This correlates well with the subjective visual analysis by two physicians blinded to scanner type, scan order or patient details, who rated the SAFOV acquisitions as being equivalent to LAFOV 1.8 ± 0.85 min acquisitions and demonstrates the clinical acceptability of such scans. As such, we are able to demonstrate a significantly improved sensitivity for a LAFOV system beyond a factor four which could be expected due to the simple extension of the FOV (106 cm vs 26.3 cm FOV). The combination of this improved sensitivity profile for the LAFOV across a larger portion of the FOV [12] and improved photon detection efficiency as a result of better scanner geometry [10] means that examinations with equivalent integral activity and SNR can be obtained in under 2 min. We highlight that these results are obtained using MRD of 85 and there is even further potential to reduce imaging time and/or injected activity once the ultra-high sensitivity mode with a MRD of 322 is clinically available.

By analogy, the effective acquisition time on the LAFOV could be maintained at the full 10 min, which remains shorter that the 16.06 min for the standard-of-care SAFOV acquisitions. Instead of reducing examination time, one could reduce injected radiopharmaceutical dose by a factor of up to 6.1x, yielding equivalent examination parameters as described above with radiopharmaceutical doses of under 40 MBq and equivalent radiation dose for the PET component of under 0.9 mSv can be reached (see Table 2). The reader is reminded that the effective examination time remains longer on the SAFOV scanner, where bed-position overlap is required, whereas the LAFOV offers a single position capture of the head to the thighs for the average adult, explaining the difference in factors between effective examination time (examination duration) and dose or acquisition time for the single bed position acquisition on the LAFOV.

Whereas previous studies report the clinical acceptability of “half-dose” protocols in LAFOV PET/CT [21], our data suggest that further and more significant activity reductions are feasible and confirm previous proof-of-concept studies showing the practicability of “low dose” protocols for LAFOV [11, 22]. In contrast to previously reported low-activity protocols proposed for digital SAFOV [24], such LAFOV protocols, are not at the cost of imaging quantification or missed target lesions, where such protocols may raise concerns about potential for clinical detriment [25]. Although EARL-compliant protocols to determine the minimum activity for lesion quantification have been published [26], the possibility for reduced radiopharmaceutical dose for PSMA- or DOTA-radioligand imaging needs to be considered, and further studies in this regard with novel LAFOV scanners are required.

In addition, while of lower visual quality compared to a standard of care SAFOV acquisition, 0.5 min LAFOV acquisitions delivered no detriment in terms of reduced target lesion-to-background ratio (TBR) or reduced lesion detectability. Such ultra-fast scans may be indicated in some circumstances, e.g. for paediatric examinations, and could simplify clinical routines involving anaesthesia, poorly compliant patients or those suffering from claustrophobia. Furthermore, although not the focus of this study, with equivalent examination doses for the PET component under 0.9 mSv, this raises the notion that PET/CT could gain popularity as a screening tool with lower radiation burden compared to some conventional screening modalities, coupled with the additional diagnostic gain of molecular imaging data.

Finally, the routine 10 min LAFOV acquisitions were of exquisite visual quality, confirmed by a blinded assessment by two experienced readers. Equivalent count densities would not be feasible on a current generation SAFOV scanner, and would require an impossibly slow theoretical table velocity of 0.11 mm/s (i.e. 9.9x slower compared to the standard 1.1 mm/s) and an impracticable effective examination time of 160 min. This serves to demonstrate that LAFOV scanners enable previously unachievable levels of image quality and quantification, even when compared to a state-of-the-art, fully digital SAFOV PET/CT system. Further improvements in imaging quality may be expected once the ultra-high sensitivity mode with a higher MRD is available. Our data suggest that LAFOV scanners can be used flexibly; according to clinical question and patient factors, ultra-low dose, ultra-fast or ultra-high-fidelity images can be obtained using a variety of tailored protocols.

One strength of this study lies in the comparison between the Siemens Biograph Vision 600 SAFOV PET/CT system and the Siemens Biograph Vision Quadra LAFOV PET/CT system, which both utilise the same detector technology, identical crystal size and identical reconstruction parameters. The Biograph Vision is already demonstrated to have excellent performance in terms of TOF sensitivity gain, equivalent to a noise reduction in the image [27], with further gains in sensitivity for the Quadra owing to the large FOV. Therefore, differences in dynamic lesion uptake notwithstanding, any resultant differences in imaging quality or lesion quantification arise predominantly as a result of the difference in scanner design, namely the FOV length, geometry and the sensitivity profile. By including the head, torso and upper thighs in the entire FOV as well as an improved axial sensitivity profile throughout the scanner compared to the SAFOV system [28], LAFOV systems demonstrate more favourable geometries compared to SAFOV systems.

The main limitation of our study is our small sample size, and this study represents the initial clinical experiences world-wide with this scanner. Although the sample size was determined in advance of the study which was adequately powered to test its hypothesis, we recognise that the sample size of 44 patients does not exclude particular clinical scenarios in which an ultra-low dose scan could result in inferior diagnostic quality and potentially impact patient management. Future studies with larger patient cohorts are required to confirm our findings and investigate other factors contributing to the total radiation exposure.

Head-to-head comparison studies inherently have an unavoidable delay between images acquired on one scanner and the next. The mean time difference between the start of the first and the second scans we report represents the time to acquire the first image, confirm the presence of target lesions, move the patient from one scanner to another and commence acquisition of the subsequent image; they are as short as the clinical service of a busy nuclear medicine clinic allows. The time between starting the first examination and the second of 47 min for ^18^F-FDG, 57 min for ^18^F-PSMA-1007 and 55 min for ^68^Ga-DOTA-TOC was as short as practicable, and scan delay as a covariate showed no relationship with lesion integral activity or SNR. Nevertheless, continued accumulation or washout of the radiotracer during this time cannot be excluded. For this reason, patients were divided into two equally sized groups undergoing the first scan on the LAFOV and SAFOV scanner respectively, minimizing any resultant bias.

Our study did not systematically test for any improved lesion detection with LAFOV systems and we highlight that all target lesions were identifiable in all LAFOV and SAFOV images. Previous studies confirm improved lesion quantification in state-of-the-art digital SAFOV systems [29] resulting in higher lesion detection [3, 7] and improved diagnostic certainty and inter-reader reliability [9] which may be the case for LAFOV scanners and for which further studies are warranted.

## Conclusion

In an intra-individual comparison in 44 patients using three common radiotracers, we are able to confirm the increased sensitivity of LAFOV scanners, both in terms of lesion quantification, image noise and subjective imaging quality. We find that such improved sensitivity means that images of equivalent quality to a state-of-the-art standard FOV digital PET/CT system can be achieved with examinations in under 2 min. By analogy, where examination time is maintained as standard, reductions in applied radiopharmaceutical dose can yield images with equivalent parameters as described and doses under 40 MBq, yielding equivalent radiation doses of under 1 mSv. As such, we demonstrate in a clinical setting that LAFOV can be used flexibly to deliver high-quality, ultra-fast or ultra-low dose examinations depending on the clinical context. The possibility for substantial reduction in minimum activities for high-sensitivity LAFOV scanners will need to be considered by future studies.
